# FDG–PET. A possible prognostic factor in head and neck cancer

**DOI:** 10.1038/sj.bjc.6600114

**Published:** 2002-02-12

**Authors:** W Halfpenny, S F Hain, L Biassoni, M N Maisey, J A Sherman, M McGurk

**Affiliations:** Department of Oral and Maxillofacial Surgery, Guy's and St Thomas's Hospitals, St Thomas's Street, London SE1 9RT, UK; Clinical PET Centre, Guy's and St Thomas's Hospitals, St Thomas's Street, London SE1 9RT, UK

**Keywords:** head and neck cancer, squamous cell carcinoma, positron emission tomography, survival

## Abstract

Previous studies have shown that high uptake of ^18^F-fluoro-2-deoxy-glucose in head and neck cancer, as determined by the standardized uptake value on positron emission tomography scan, was associated with poor survival. The aim of this study was to confirm the association and to establish whether a high standardized uptake value had prognostic significance. Seventy-three consecutive patients with newly diagnosed squamous cell carcinoma of the head and neck underwent a positron emission tomography study before treatment. Age, gender, performance status tumour grade, stage, maximal tumour diameter and standardized uptake value were analyzed for their possible association with survival. The median standardized uptake value for all primary tumours was 7.16 (90% range 2.30 to 18.60). In univariate survival analysis the cumulative survival was decreased as the stage, tumour diameter and standardized uptake value increased. An standardized uptake value of 10 was taken as a cut-off for high and low uptake tumours. When these two groups were compared, an standardized uptake value >10 predicted for significantly worse outcome (*P*=0.003). Multivariate analysis demonstrated that an standardized uptake value >10 provided prognostic information independent of the tumour stage and diameter (*P*=0.002). We conclude that high FDG uptake (standardized uptake value>10) on positron emission tomography is an important marker for poor outcome in primary squamous cell carcinoma of the head and neck. Standardized uptake value may be useful in distinguishing those tumours with a more aggressive biological nature and hence identifying patients that require intensive treatment protocols including hyperfractionated radiotherapy and/or chemotherapy.

*British Journal of Cancer* (2002) **86**, 512–516. DOI: 10.1038/sj/bjc/6600114 www.bjcancer.com

© 2002 Cancer Research UK

## 

Squamous cell carcinomas (SCC) of the head and neck are a distinct group of neoplasms with an unpredictable clinical behaviour. The stage of the disease is currently the most important prognostic parameter and depends partly on local invasion but more to cervical node status. The biology of each tumour is important to outcome, for even in advanced disease (stages 3 and 4) a third of patients can be cured. However, at present these patients cannot be distinguished.

In positron emission tomography (PET) the most commonly used radiopharmaceutical is ^18^F-fluoro-2-deoxy-glucose (FDG), an analogue of glucose, which is metabolized in normal, and neoplastic tissues in proportion to the rate of tissue metabolism. This tracer is particularly useful in Oncology (Conti and Strauss, 1991; Minn *et al*, 1995), because it exploits the increased glucose metabolism present in the majority of malignant tumours (Warburg, 1956).

FDG–PET has already been applied to staging and follow-up of patients with head and neck cancer (Rege *et al*, 1994; Braams *et al*, 1995), and Minn *et al* (1997) have shown that high uptake as determined by the standardized uptake value (SUV) was associated with poor survival. However it is not clear whether a high SUV simply reflects the stage of disease or is an independent predictor of survival. The aim of this study was to investigate the relationship of SUV values to outcome in head and neck cancer.

## MATERIALS AND METHODS

### Patients and treatment

Between February 1993 and September 1998, 73 consecutive patients with newly diagnosed Squamous cell carcinoma of the head and neck underwent an FDG–PET evaluation before treatment. Diabetic patients (eight patients) and those with incomplete data (seven patients) were excluded from the study. Diabetics were excluded because of their altered glucose metabolism and its effect on SUV (Hallett *et al*, 2001). Incomplete data included stage and/or SUV leaving a total of 58 patients suitable for analysis.

All patients had panendoscopy and CT scans of the head and neck and were staged according to the UICC TNM classification (Hermanek and Sobin, 1992). The average diameter of the primary tumour was calculated from clinical inspection and the CT scan. Patient characteristics are listed in Table 1

**Table 1:**
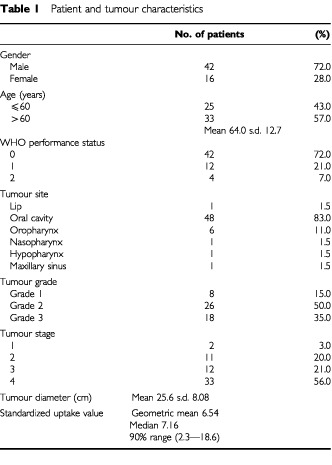
Patient and tumour characteristics

.

All patients were treated with curative intent. Fifty-three patients (91%) underwent surgery as the primary treatment modality, and of these patients 75% received postoperative radiotherapy. Five patients received radiotherapy alone. Of the patients undergoing surgery 38 had local excision with a neck dissection and free tissue transfer reconstruction, 10 local excision and a neck dissection and five local excision alone. Patients were followed up at 2 monthly intervals with a median follow-up of 39 months (range 4–75 months).

### FDG–PET study

The patient fasted for 6 h prior to the PET study and a blood glucose evaluation proceeded the test. FDG was synthesized with radiochemical purity >99% and 350 MbQ were injected intravenously. After a 30–60 min uptake period images were acquired in 2D mode on an ECAT 951R scanner (Siemens/CTI, Knoxville, TN, USA). Emission data were acquired at 15 min per bed position (two positions) each of 31 slices over a 10.6 cm axial field of view. A transmission scan using Germanium 68 source was obtained for attenuation correction for 10 min following the emission. Images were reconstructed using filtered back projection (Hann filter, cut-off frequency 0.4) and viewed as coronal, transaxial and sagittal slices with a spatial resolution of 12 mm.

Standard uptake values (SUV) of the main lesion were calculated using the attenuation corrected image. One observer positioned a 4.5 mm region of interest over the maximum pixel in the lesion and the average of two perpendicular diameters of the lesion was noted. The SUV was calculated using the formula: tissue concentration of FDG measured by PET divided by the injected dose divided by body weight. No correction was made for glucose or partial volume.

### Statistical analysis

Statistical analysis was performed using a standard software package (Stata: Release 7.0, Stata Corp., College Station, TX, USA). Standard diagnostics showed that age and tumour diameter had a normal distribution and SUV a log-normal. For age and diameter, mean and s.d. are given. For SUV, geometric mean, median and 90% range (5th to 95th centile). For categories, percentages are used.

The effect of tumour diameter and stage on SUV was examined by linear regression on the log of SUV. Analysis of SUV initially examined survival within SUV subgroups that were defined by the quartiles of SUV distribution. A log rank test was used to determine a statistically significant cut-off value of 10, which was used for survival analysis.

Survival times were calculated from the time of the PET study. The eight intercurrent deaths were all regarded as clearly unrelated to head and neck cancer and were treated as censored at time of death. A stepwise Cox regression analysis was performed for recognized risk factors: first unadjusted (‘univariate’) and then adjusting (‘multivariate’) for the main predictors (stage tumour diameter and SUV). Due to the small number of patients with stage 1 disease they were combined with the stage 2 patients for analysis. For similar reasons those patients classified as performance status 2 were combined with the performance status 1 group. Survival curves were obtained by the Kaplan–Meier method. Robust standard errors were used to correct for possible unequal variances and any residual divergence from normality. A *P* value of less than 0.05 was considered significant.

## RESULTS

All primary tumours were correctly identified on the PET scans. The mean tumour diameter was 25.60 mm (s.d. 8.08*)*. Outcome evaluation showed 28 out of 58 patients (48%) were dead by the end of the study period: 20 (72%) from disease and eight (28%) from other causes (myocardial infarction (four), stroke (three), prostate cancer (one)). The stage of disease at initial presentation of those who died of head and neck cancer was: stage 2 (*n*=1), stage 3 (*n*=3) and stage 4 (*n*=16).

**Figure 1:**
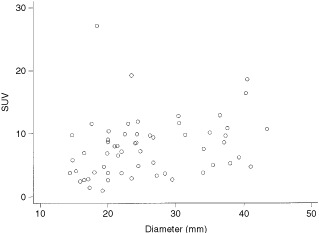
Scattergram of SUV with tumour diameter.

The median SUV for all primary tumours was 7.16 (90% range 2.30 to 18.60). Stage was a more important predictor of SUV than tumour diameter ( Figures 1

and 2

**Figure 2:**
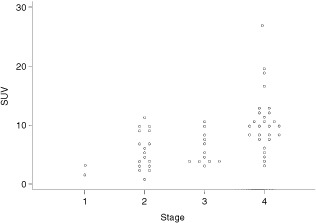
Scattergram of SUV with stage.

). An increase in tumour diameter of 1 cm resulted in only a 1.06 times rise in SUV (Ratio 1.06, 95% CI 0.8–1.3 *P*=0.55), compared to a four-fold rise in moving from stage 1 to stage 4 disease (Ratio 4.17, 95% CI 2.4–7.4 *P*=0.004).

In univariate survival analysis high stage (*P*=0.015), increasing tumour diameter (*P*=0.041) and SUV >10 (*P*=0.003) were the main negative predictors of survival ( Table 2

**Table 2:**
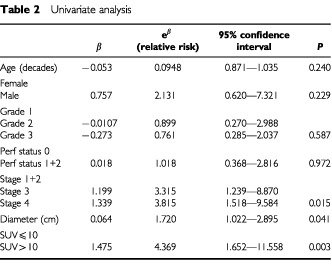
Univariate analysis

). The Kaplan–Meier survival curves are shown in Figures 3

**Figure 3:**
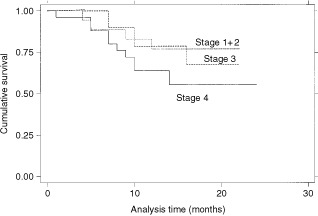
Kaplan–Meier survival estimates, by stage.

, 4

**Figure 4:**
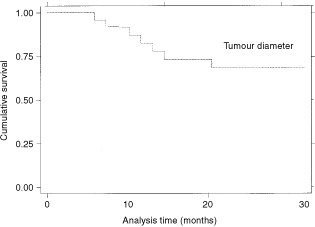
Kaplan–Meier survival estimate, by tumour diameter.

and 5

**Figure 5:**
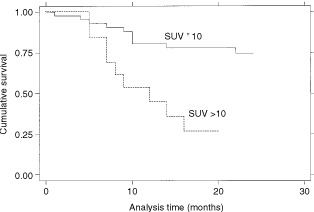
Kaplan–Meier survival estimates, by SUV10.

.

In multivariate analysis the joint effect of stage, tumour diameter and SUV was examined to determine whether SUV had prognostic significance beyond that provided by stage and tumour diameter ( Table 3

**Table 3:**
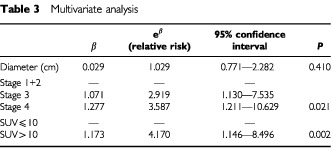
Multivariate analysis

). Patients with an SUV >10 had a significantly poorer prognosis (*P*=0.002). The relative risk was increased for patients with high stage disease ( Figure 6

**Figure 6:**
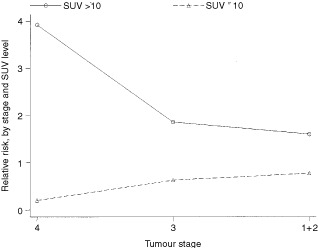
Relative risk of death by SUV and stage.

).

## DISCUSSION

The role of PET–FDG in the management of squamous cell carcinoma (SCC) of the head and neck continues to evolve and its full potential has yet to be established. It has been applied to staging and surveillance of patients with head and neck SCC (Rege *et al*, 1994; Braams *et al*, 1995), but a more promising observation is that high FDG uptake in head and neck SCC is associated with poor survival (Minn *et al*, 1997). A similar association has also been demonstrated for other malignancies such as brain, lung and malignant lymphomas (Patronas *et al*, 1985; Okada *et al*, 1992; Ahuja *et al*, 1998), where it appears that the prognostic value of a high SUV may act independently of tumour stage.

The role of SUV as a prognostic factor has been extensively studied in non-small cell lung cancer (NSCLC) which consists largely of patients with SCC. This tumour type has a very similar biology to SCC of the head and neck, and smoking is a common aetiological agent. It is not surprising therefore that the present results mirror those for lung cancer, where SUV has proved to be a significant prognostic indicator (Ahuja *et al*, 1998; Saunders *et al*, 1999; Vansteenkiste *et al*, 1999).

Vansteenkiste *et al* (1999) suggested a SUV of seven, and Ahuja *et al* (1998) one of 10 as a cut-off value that distinguishes good and bad prognostic groups, although there is probably a range of 6–11 where prognosis deteriorates. Previous studies in head and neck cancer have found SUV important in differentiating benign and malignant disease but were unable to define a cut-off value (Nowak *et al*, 1999). In the present study a SUV >10 proved to be an independent marker of poor prognosis and this is consistent with results obtained for lung cancer.

Uptake time is important in sarcomas (Lodge *et al*, 1999) and possibly testicular cancer (Hain *et al*, 2000). The optimal time allowed for FDG uptake in squamous cell carcinoma of the head and neck is unknown. The uptake period was consistent throughout this study (30–60 min). Where differences have been seen the uptake has varied over a period of hours rather than 30–60 min.

The association of high FDG uptake with poor survival may be related to several factors. Firstly, most patients with advanced disease present with large tumours, and FDG uptake may simply reflect the larger tumour burden in these patients. Indeed some of the lung studies have shown that size as well as SUV has prognostic value (Saunders *et al*, 1999; Vansteenkiste *et al*, 1999). However in the current study increasing tumour diameter had little effect on FDG uptake (SUV), suggesting that tumour burden alone cannot account for these findings.

Secondly, it has been proposed that the abnormal carbohydrate metabolism seen in the cachectic state is responsible for the disproportionate glucose uptake, in tumours of patients with advanced malignancies (Tayek, 1992). An association has been demonstrated between FDG uptake and cellular proliferation and/or tumour growth rate in a number of cancers including head and neck, lung tumours and non-Hodgkin's lymphoma (Minn *et al*, 1988, 1997; Haberkorn *et al*, 1991).

Thirdly, Kitagawa *et al* (1999) found that SUV value in SCC of the head and neck pre-treatment predicted response to chemo/radiotherapy, with tumours demonstrating higher SUV having greater treatment resistance. Together these observations provide tentative evidence that the rate of glucose metabolism in SCC may reflect adverse tumour biology and survival. If this proves correct, FDG uptake represents one of the few objective measures of tumour malignancy.

In this study high FDG uptake (SUV >10) proved to be a marker for poor outcome in SCC of the head and neck, particularly in those patients with stages 3 and 4 disease. SUV increased with stage, but multivariate analysis suggests SUV was an independent predictor of survival, and not dependent on stage. Therefore patients with tumours that are more active metabolically, as demonstrated by FDG–PET imaging, should be considered at high risk for relapse, regardless of clinical stage at presentation. This is similar to results obtained in lung cancer where both PET staging and SUV are currently the best predictors of survival, apart from operative stage (Saunders *et al*, 1999). If these findings are substantiated it will allow the clinician to distinguish those tumours with a more aggressive biological nature, and hence identify those patients that require intensive treatment protocols, including hyperfractionated radiotherapy and/or chemotherapy.
